# On‐Surface Driven Formal Michael Addition Produces *m*‐Polyaniline Oligomers on Pt(111)

**DOI:** 10.1002/anie.202009863

**Published:** 2020-10-12

**Authors:** Nerea Ruiz del Árbol, Carlos Sánchez‐Sánchez, Gonzalo Otero‐Irurueta, José I. Martínez, Pedro L. de Andrés, Ana C. Gómez‐Herrero, Pablo Merino, Marten Piantek, David Serrate, Paolo Lacovig, Silvano Lizzit, José Alemán, Gary J. Ellis, María F. López, José A. Martín‐Gago

**Affiliations:** ^1^ ESISNA Group, Materials Science Factory Institute of Materials Science of Madrid (ICMM-CSIC) Sor Juana Inés de la Cruz 3 28049 Madrid Spain; ^2^ Centre for Mechanical Technology and Automation (TEMA) University of Aveiro 3810-193 Aveiro Portugal; ^3^ Instituto de Ciencia de Materiales de Aragón CSIC-Universidad de Zaragoza 50009 Zaragoza Spain; ^4^ Elettra—Sincrotrone Trieste S.C.p.A. Strada Statale 14 km 163.5 34149 Trieste Italy; ^5^ Organic Chemistry Department, Módulo 1 Universidad Autónoma de Madrid 28049 Madrid Spain; ^6^ Polymer Physics Group Institute of Polymer Science and Technology (ICTP-CSIC) Juan de la Cierva 3 28006 Madrid Spain

**Keywords:** coupling reactions, DFT, nc-AFM/STM, on-surface synthesis, Polyaniline

## Abstract

On‐surface synthesis is emerging as a highly rational bottom‐up methodology for the synthesis of molecular structures that are unattainable or complex to obtain by wet chemistry. Here, oligomers of *meta*‐polyaniline, a known ferromagnetic polymer, were synthesized from *para*‐aminophenol building‐blocks via an unexpected and highly specific on‐surface formal 1,4 Michael‐type addition at the *meta* position, driven by the reduction of the aminophenol molecule. We rationalize this dehydrogenation and coupling reaction mechanism with a combination of in situ scanning tunneling and non‐contact atomic force microscopies, high‐resolution synchrotron‐based X‐ray photoemission spectroscopy and first‐principles calculations. This study demonstrates the capability of surfaces to selectively modify local molecular conditions to redirect well‐established synthetic routes, such as Michael coupling, towards the rational synthesis of new covalent nanostructures.

## Graphical abstract

A novel, on‐surface synthesis of oligomers of *meta*‐polyaniline from *para*‐aminophenol precursors by an unexpected formal 1,4 Michael‐type addition at the *meta* position is reported. Experimental and theoretical results show that the reaction proceeds via a highly specific interaction between the precursor and the Pt surface that induces the reduction of the aminophenol molecule, opening a door to the rational synthesis of new molecular structures.

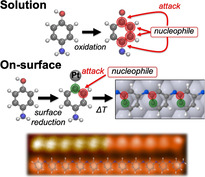

## Introduction

**Scheme 1:**
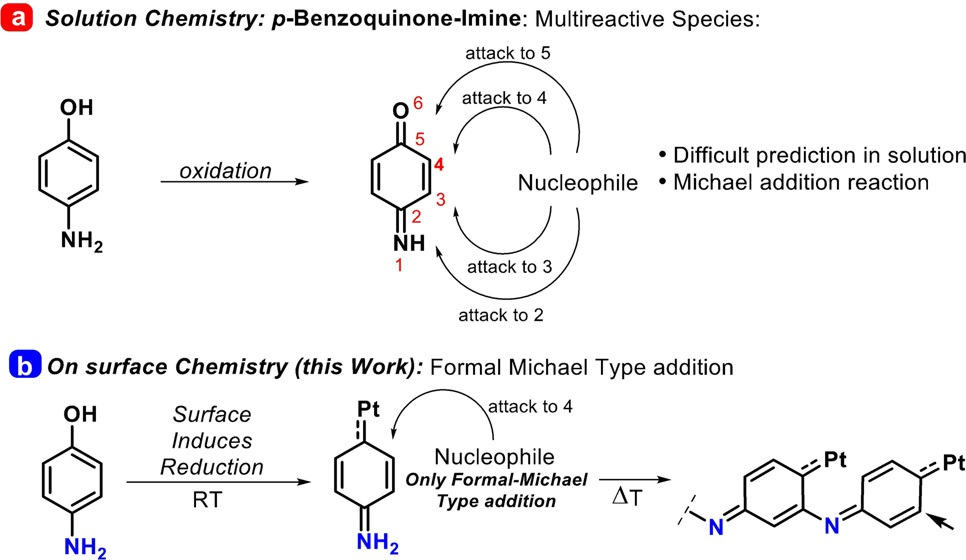
Comparison of the Michael addition a) in solution and b) on a surface (this work). Dashed lines in panel (b) indicate interaction with a surface atom.

Quinone and quinone imines in general, and 1,4‐benzoquinone monoimine in particular, are very useful building blocks for the synthesis of a wide variety of compounds,^[1]^ from natural products to polymers, with a broad range of applications. Despite the a priori structural simplicity of such molecules, they are characterized by a lack of control of the reactive sites due to the electrophilic character of the different positions on the ring (see positions 2, 3, 4, and 5 in equation a, Scheme 1).^[2]^ Therefore, in order to target the synthesis of a specific molecular structure, it is crucial to find a reaction that induces selectivity. In this work, we show that a route based on the well‐known Michael addition,[^3^, ^4^] complemented with the unique properties of metal surfaces, generates high selectivity towards nucleophilic attack at site 4 (see Scheme 1, panel b), such that starting from simple *para*‐aminophenol (*p*‐AP) precursors we obtain *meta*‐coupled oligomers of polyaniline (PANI).

Conjugated polymers such as PANI are a family of polymers that have attracted huge scientific and technological attention during the last decade.[^5^, ^6^, ^7^] The emeraldine base form is generally regarded as the most useful, since it becomes electrically conducting upon simple protonation of the imine nitrogen atoms with an acid. Indeed, one of their most important properties is the reversibility of electronic character and the modification of electrical conductivity through doping (protonation) and undoping (deprotonation) treatments that, combined with their relatively facile processability and good environmental stability, make them interesting materials for a wide range of applications.^[8]^ Additionally, there is growing recent interest in PANI chains polymerized in the *meta*‐position (*m*‐PANI), since its well‐known room‐temperature ferromagnetic behaviour[^9^, ^10^] has become of interest for tuning electronic and magnetic states, spin transport and spin interactions that may have important implications for applications in organic conductors and spintronics^[11]^ and as possible precursor materials for the development of carbon magnets.^[12]^ Albeit there are several synthetic routes to *m*‐PANI homopolymers[^13^, ^14^, ^15^, ^16^] or *m*‐PANI/*p*‐PANI copolymer‐like structures,[^17^, ^18^] these reactions can be challenging, requiring multistep processes to obtain adequately pure materials.

On‐surface synthesis has consolidated as a unique strategy to obtain unprecedented low‐dimensional carbon‐based nanomaterials with atomic precision showing intriguing properties.^[19]^ Among the variety of reactions successfully tested on surfaces, Ullmann coupling remains the preferred methodology for inducing on‐surface homo‐ and hetero‐coupling of individual molecular precursors. With such strategies, diverse one‐dimensional nanostructures including conducting polymers and atomically precise graphene nanoribbons (GNRs) have been recently synthesized.[^20^, ^21^, ^22^, ^23^, ^24^, ^25^, ^26^, ^27^] However, this methodology presents several drawbacks, such as surface poisoning by the detached halogen, or the need to dispose of halogen modified precursors. Although methods to reduce these limitations have been described,^[28]^ alternative strategies, based on innovative reaction mechanisms, are needed. Moreover, one of the most important merits of on‐surface synthesis is the ability to open new reaction pathways that are inaccessible via solution‐based organic chemistry, thanks to the particular catalytic role played by surfaces. Thus, in this work we describe a mechanism for surface‐induced selectivity in a formal 1,4 Michael‐type addition, without the need for a halogen substituent. To illustrate this, we have used platinum, a metal well‐known for its strong dehydrogenation capability.[^30^, ^31^] Specifically, we show that the unique reaction pathway induced by the Pt(111) surface leads to the formation of linear *meta*‐polyaniline (*m*‐PANI) oligomers from a precursor monomer functionalized at *para* positions (*p‐AP*). We demonstrate that the interaction with the platinum surface not only drives the reaction mechanism at a specific site but also aligns the growing oligomers along the crystallographic directions of Pt, self‐limiting the length of the oligomers due to stress accumulation. These type of reactions, where the position of attack differs from the activated site, are very rare, thus very interesting as they open new reaction pathways.

The reaction mechanism is unveiled by a combination of in situ advanced microscopy and spectroscopy techniques, including non‐contact atomic force microscopy (nc‐AFM), scanning tunneling microscopy (STM) and spectroscopy (STS), synchrotron‐based X‐ray photoemission spectroscopy (XPS), and theoretical calculations. Our results unequivocally demonstrate the chemical transformation of the precursors upon annealing and the chemical structure of the synthetized oligomers. Moreover, these techniques allow us to determine the electronic structure of the oligomers, which is distinguished by the presence of unoccupied localized electronic states in the chains. This work is a clear example demonstrating that chemical routes that are complex or inefficient in solution‐based chemistry can be accomplished via on‐surface chemistry, opening the door to new approaches for the synthesis of defined supramolecular structures.

## Results and Discussion

**Figure 1:**
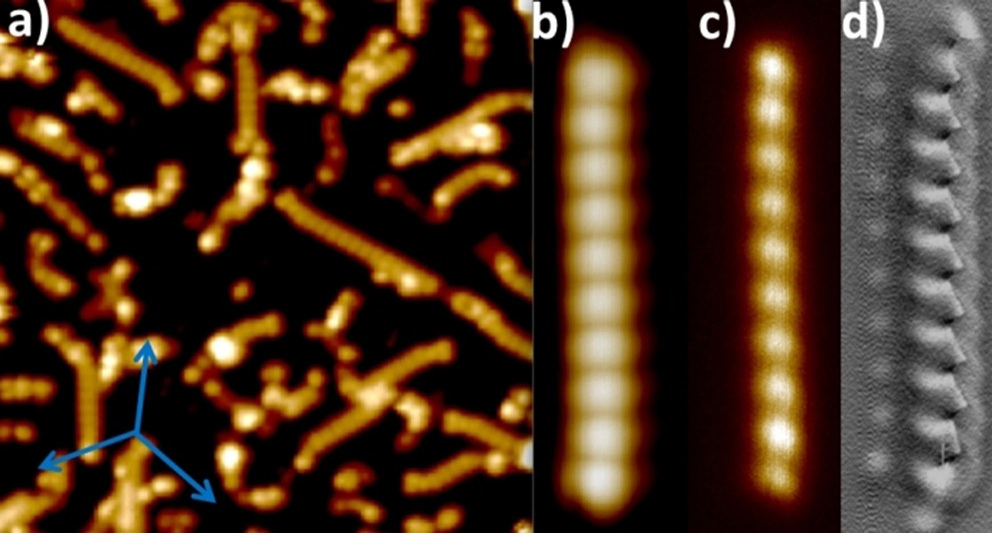
Low Temperature (5 K) STM/nc‐AFM images of the PANI oligomers that are formed after the evaporation of *p*‐AP molecules on Pt(111) on the hot surface. a) Overview (20×20 nm^2^) constant current STM image upon deposition at 525 K, measured at 0.8 V and 50 pA. Blue arrows represent the main crystallographic directions of the Pt(111) single crystal. b) (6×2) nm^2^ constant current STM image of oligomers formed at 475 K, measured at 0.1 V and 20 pA. c) (6×2) nm^2^ constant height STM image recorded after the (b) image (regulated over the oligomer center at 0.1 V, 20 pA and Δz=−0.5 Å). d) (6×2) nm^2^ constant height nc‐AFM frequency shift image of the same single oligomer chain of polyaniline recorded with a CO‐functionalized tip. This chain is formed from 10 molecules of *p*‐AP (regulated at 0.1 V and 20 pA, Δz=−2.7 Å at the central lobe of the polymer).

The interaction of *p*‐AP molecules on a Pt(111) surface at room temperature has been previously described whereby the formation of partially ordered molecular layers was found.^[34]^ Figure 1 a shows a typical STM/nc‐AFM image obtained after depositing *p*‐AP molecules on a Pt(111) surface at a temperature of around 525 K. This image shows some individual isolated molecules that have not reacted together along with two types of elongated structures. On the one hand, curved chains with an inhomogeneous appearance are observed. On the other, homogenous linear chains can be seen, made of individual links ranging from 3 to 12 units. The latter chains are oriented at 30° with respect to the main crystallographic directions of Pt(111) and show an average internal periodicity of 4.6±0.1 Å and, a width of 7 Å (values obtained from statistical analysis of hundreds of oligomers from STM images like that in Figure 1 a). Figures 1 b–d show details of a single linear chain composed of 10 monomers formed upon deposition at 475 K. In the constant current mode image (Figure 1 b), the chain appears as a periodic repetition of bright elliptical lobes, whilst in the constant height image (Figure 1 c) the lobes have a rounded shape. Importantly, these maxima present an identical appearance in all the oligomers observed, suggesting that each has the same local structure. In order to improve the intramolecular resolution, a frequency‐shift (Δ*f*) image of the tip‐oligomer force interaction was performed on the same chain using a CO functionalized tip (Figure 1 d). In this image, the lobes are resolved as tilted hexagonal shapes, which we attribute to the phenyl ring of each building block. In addition, some rounded protrusions appear when the tip is close to the sample. At present, we do not have a clear interpretation of the nature of these protrusions. It can also be seen that the top and bottom edges of the frequency shift image show protrusions that differ in appearance, which is in good agreement with the expected differences between the chain terminations, as discussed below.

**Figure 2:**
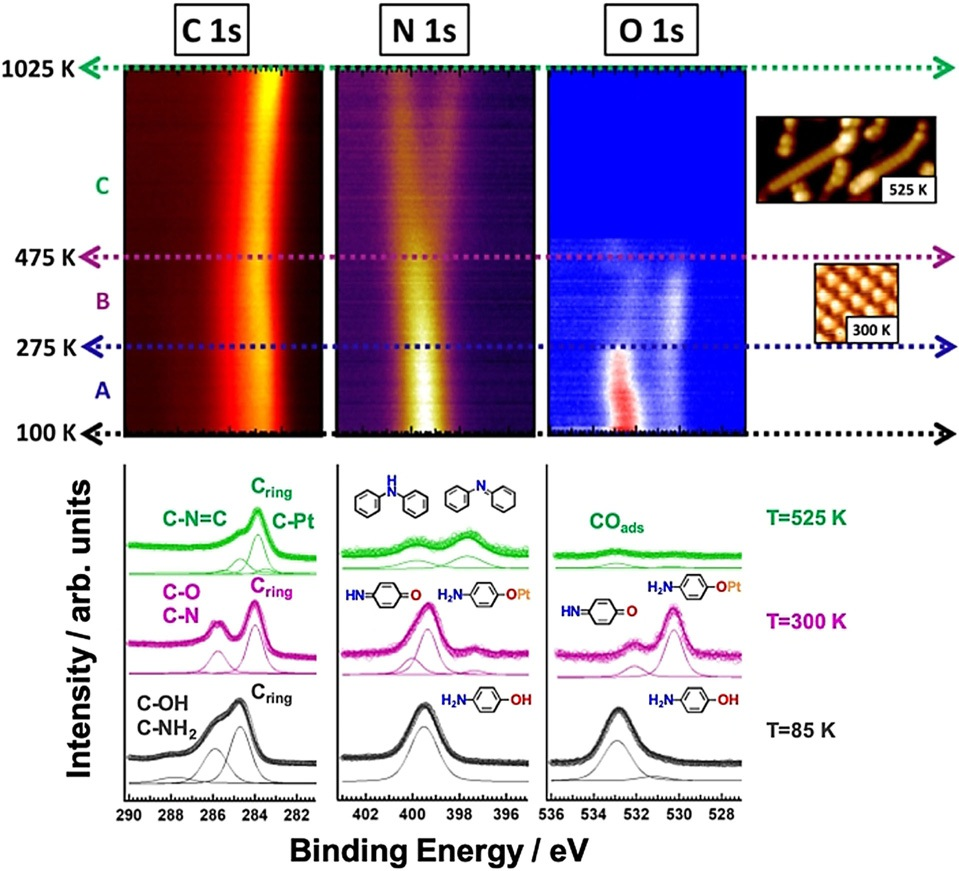
Top: “Fast” C 1s, N 1s and O 1s XPS core levels as a function of the annealing temperature after evaporation of *p*‐AP on Pt(111). The N 1s “fast” XPS scale ranges from the less intense signal in black to the more intense in yellow, while for O1s ranges from the less intense in blue to the more intense in red colour. Three different regions (A, B, C) are observed. At right side, characteristic STM images for these regions. Lower part: Characteristic high‐resolution C 1s, N 1s and O 1s XPS core levels spectra recorded after depositing at high temperature and cooling at 120 K at the three different regions (A, B, C) observed in the top panel. The blue spectra correspond to a molecular multilayer, while the purple and green ones are associated to monolayer or submonolayer coverage due to molecular desorption during sample annealing.

To unveil the chemical nature of these oligomers, we have studied the thermal behaviour of the core levels associated to the molecules using synchrotron radiation‐based XPS following two different approaches. On the one hand, we have performed a “fast” (real‐time) XPS of the C 1s, N 1s, and O 1s core level peaks while ramping the sample temperature from 120 K to 1025 K. The results are plotted in the 2D representation shown in the top panel of Figure 2.^[35]^ This type of experiments allow for a one‐shot qualitative understanding of the chemical transformations experienced by the molecule during annealing. Although the absolute temperatures are usually overestimated in this type of measurements due to kinetic considerations (the system has no time to thermalize), these pictures are very useful to determine at a glance the temperature regions where chemical transformations take place. Three different phases can be distinguished in the “fast” XPS, and detailed high‐resolution XPS spectra have been recorded at each of the selected regions (lower panel in Figure 2). The bottom spectra in Figure 2 (black line) correspond to a multilayer of *p*‐AP molecules recorded at 80 K, used as a reference for the core level binding energies (BE). The BE obtained for the amino group, the alcohol group, and the carbon ring are 399.5 eV, 532.8 eV, and 284.7 eV, respectively. These values appear shifted by about 0.4 eV to higher BE than the values reported in literature for adsorbed molecules[^36^, ^37^, ^38^] due to the presence of a multilayer thick enough to prevent a contribution from the contact layer, which experiences a final state screening by the Pt substrate.

In the temperature range from 120 K to 275 K (region A), most of the molecules preserve their canonical structure, with a minority of them undergoing the initial stages of the dehydrogenation of the alcohol groups. The second phase occurs between 275 K and 475 K (range B) and corresponds to STM images showing individual molecules. The intensity of all peaks slightly decreases, but the total stoichiometry derived from XPS quantitative analysis (see Supporting Information) indicates that neither N nor O atoms are removed from the molecules. However, full dehydrogenation of the hydroxyl moieties leads to the appearance of new components in the O 1s core level peak. Most of the species present a strong interaction with the Pt surface (new component at 530.3 eV for O 1s) through activated site **6** (see Scheme 1), while the rest adopt a quinone form as suggested by the binding energy of the related peak (532.3 eV).[^38^, ^39^, ^40^] On the other hand, most of the amine groups remain intact (BE of 399.35 eV for N 1s) while a minority of the molecules present partially and fully dehydrogenated nitrogen species (peaks with BE of 399.9 eV and 397.6 eV, respectively).^[41]^

Finally, at temperatures around 475 K (range C), linear chains are formed through covalent coupling between the activated molecules, as can be observed in the STM images of Figure 1. XPS reveals that oxygen is fully removed from the surface, leaving an insignificant contribution at 533 eV, due to adventitious oxygen (note that XPS measurements are carried out at 120 K). On the other hand, two contributions for N 1s are observed, located at 399.8 and 397.6 eV, which suggest that the linear chains are comprised of aryl units linked by nitrogen atoms. In different studies related with polyanilines, the core level associated with benzenoid amine varies in the range of 399.9–399.3 eV and for quinone imine between 398–398.9 eV.[^42^, ^43^, ^44^] The value of 397.6 eV corresponding to the quinone imine is lower than expected, but this difference can be attributed to charge redistribution due to the interaction with the metal surface. The C 1s core‐level peak presents three components at 283.4 eV, 283.9 eV, and 284.8 eV, that can be attributed to the C_ring_‐Pt bonds, C ring and azo‐linkages, respectively. Therefore, the XPS analysis of N 1s and C 1s core levels indicates that the oligomers depicted in Figure 1 present a PANI structure, with a co‐existence of imine and secondary amine linkages.

Region C in the “Fast” XPS data in Figure 2 shows an important decrease in the N 1s intensity (much less pronounced in the C 1s peak) and an increasing peak shift upon increasing temperature. These effects can be rationalized in terms of a gradual temperature‐induced decomposition of the oligomers and a concomitant loss of N atoms as a consequence of the interaction with the metal surface (see corresponding shift of the C 1s peak towards lower BE characteristic of C‐metal interactions). Interestingly, at the extreme temperature of 1025 K, the N 1s presents two components at approximately 400.5 eV and 398.3 eV. The former is attributed in literature to N atoms embedded in a graphene lattice,[^45^, ^46^] whilst we assign the latter to the remaining oligomers that partially preserve their structural integrity. Decomposition of azo‐derived molecules at high temperature on platinum surfaces and formation of small graphene areas has already been reported (see Supporting Information).[^30^, ^47^]

**Figure 3:**
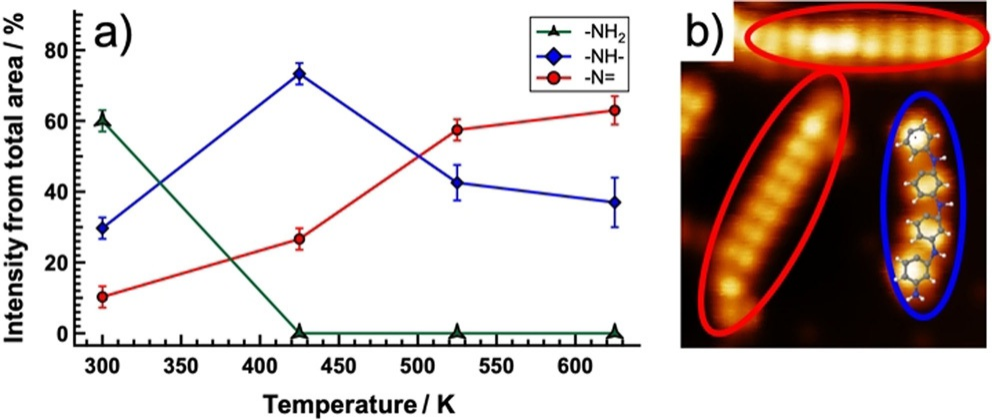
a) Evolution of the amine (‐NH_2_) termination, secondary amine (‐NH‐), and imine (‐N=) linkages components at the N 1s core‐level peak as a function of temperature. Data obtained from N1s integration b) Constant height STM image showing the two kinds of oligomers more frequently found on the surface. Over the curved one a plausible scaled model has been superimposed. STM parameters: (6×7 nm^2^). Feedback open at 0.15 V and 4.0 nA.

Figure 3 a shows the thermal evolution of the normalized intensity of the three components of the N 1s photoemission peak, as extracted from a detailed deconvolution of the XPS peaks. It can be observed that at RT the NH_2_ moiety predominates (60 %), as is expected for the adsorbed molecule, together with lower contributions of ‐NH (30 %) and ‐N= (10 %). As previously mentioned, from the STM images two types of elongated molecular structures can be distinguished on the surface: linear (discussed above) and curved structures. Figure 3 b shows a STM image of a zone where both structures coexist. The former (highlighted by red ellipses) are assigned to fully oxidized PANI oligomers based on imine links, while the latter structure (highlighted by blue ellipses) would correspond to the reduced form of PANI formed by ‐NH‐ (amine) links. This assignment is based on the experimental observation from STM images of an increasing proportion of linear vs. curved oligomers with temperature, the former being prevalent at 625 K (see Supporting Information). We rationalize this by considering a surface oxidation of the amine links into imine links. Moreover, these findings are corroborated by the structural models inferred from theoretical calculations, as we will argue below.

**Figure 4:**
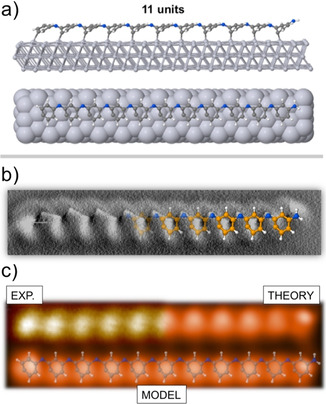
a) Top and side view of the DFT‐optimized model for the m‐PANI of 11‐units oligomer on Pt(111). b) DFT‐optimized model superimposed on the nc‐AFM image of Figure 1 d. c) Simulated STM image (Keldish‐Green's functions formalism) at constant‐current regime (0.1 nA) for the 11‐units oligomer on Pt(111). Computed image has been obtained by integrating the STM current from the Fermi energy up to a bias of +0.75 V. The schematic oligomer geometry has been superimposed to facilitate the understanding of the simulated image.

Taking into account the aforementioned information provided by STM/AFM and XPS, one can conclude that *p*‐AP molecules on Pt(111) lead to the formation of PANI oligomers above 475 K. A first approximation would be to assume that the polymerization takes place in a *para* configuration (positions 1–5 in Scheme 1), as oxygen has been cleaved from the carbon ring. However, a detailed analysis of the experimental results indicates that this cannot be the case. The periodicity within the oligomers, represented by the distance between adjacent phenyl rings for free *p*‐PANI, is expected to be 5.2 Å, as shown by accurate gas phase DFT calculations and elemental arguments based on the well‐accepted dimensions for these molecules. However, we have found an experimental periodicity of 4.6±0.1 Å, leaving a discrepancy of 13 %, which is too large to be accommodated by surface‐induced molecular distortions. Moreover, Figure 1 shows that all repeat units within each oligomer are equivalent, whereas the *p*‐PANI structure would present a zig‐zag configuration resulting from out‐of‐plane alternate tilting of the phenyl rings. Therefore, to determine the atomic structure of the oligomer, we have performed an extensive number of DFT calculations and the optimization of different plausible structures. A best candidate that fits well with both XPS and STM/AFM data has been found and is presented in Figure 4 (see Supporting Information for the rest of the structures). This model suggests that the oligomers adopt an *m*‐PANI configuration that is stabilized along the [2$\bar{1}$ $\bar{1}$ ] surface direction (and equivalent symmetry‐related directions), in agreement with the experimental observation of Figure 1 a, whilst maintaining a significant interaction with the surface through position **5** of the phenyl ring (see Scheme 1). In fact, this interaction is reflected in the short adsorption distance between **C5** and Pt surface, 2.1 Å, indicating covalent bonding of C atom with the surface, and in the C 1s core level signal where a small peak at 283.4 eV appears (one sixth of the C intensity, corresponding to the expected ratio between C species). As a consequence, the different phenyl rings present an in‐phase torsion angle ranging between 20° and 30° that is responsible for the shape of the aryl repeat units observed in Figure 1 d. Finally, the Pt atoms connecting with these lowest C atoms exhibit a perpendicular *out‐of‐plane* buckling of around 0.15 Å, confirming a significant build up interaction, whereas the rest of the Pt atoms maintain the *in‐plane* configuration.

Another important result derived from the calculations is that the oligomers are not commensurate with the Pt surface. The preferred model (Figure 4) exhibits a computed periodicity of 4.62–4.68 Å (to be compared with the experimentally measured average periodicity of 4.6±0.1 Å), which does not fit with the Pt‐Pt distance of 4.81 Å along the [2$\bar{1}$ $\bar{1}$ ] high‐symmetry direction. This fact implies that each arylamine unit added to the chain accumulates stress corresponding to a longitudinal mismatch of around 0.2 Å per repeat unit with respect to the substrate. The experimental observation of a maximum length of 11 units suggests that the maximum accumulative stress that the oligomer can assimilate corresponds to a mismatch of about 20 %, which is a considerable value considering the rigidity of the imine bond.

Panel c) of Figure 4 shows the computed STM image in constant‐current mode. The correlation between simulated and experimental images is excellent, both showing a linear sequence of rounded protrusions, with one of the extremes presenting a different appearance due to the presence of the NH_2_ group. The superposition of the geometrical model over both the nc‐AFM image (Figure 4 b) and the theoretical STM image (Figure 4 c) allows the assignment of each rounded protrusion to the aryl units in the chain, except the one closest to the NH_2_ extreme, where the intensity of the ring is attenuated and a smaller protrusion associated to the C connected to the NH_2_ group appears, as it is observed in the experiment. The Δ*f* image in Figure 4 b provides a clear view of the asymmetry in the terminal groups of the oligomer.

As we have mentioned, *m*‐PANI chains are not commensurate with the Pt substrate, regardless of their length. To understand the reasons, we explore the energetics and electronic properties of the oligomer adsorbed on the surface. In the proposed model of Figure 4, monomers are linked by C−N=C units, which have bond lengths between C and N of 1.33 Å and Pauling bond orders of around 1.5, reflecting the resonance between a single and a double bond. Such a configuration stores −5.3 eV per C−N bond (gas phase) and results in a rigid configuration, which does not accommodate rotations easily. Further, it dictates a length for the repeating unit of the polymer that is 0.2 Å shorter, which prevents commensuration with the underlying Pt lattice. Therefore, the interplay between its internal energy and the interaction with the substrate results in stress for the growing polymer chain. To complete our understanding on the formation of the oligomer, we have performed additional simulations; it is interesting to briefly discuss the case where monomers are joined via C‐NH‐C units (e.g. configuration II in Figure S1). Here, C−N bond lengths extend to 1.39 Å, and it stores −3.9 eV (gas phase), with a bond order close to 1. Such a configuration is more flexible than the previous one and it generates a repeat unit in the polymer that can commensurate better with the substrate. However, the commensurability only increases the interaction energy per monomer with the substrate from 1.3 to 1.95 eV (*Δ*=−0.65 eV), which explains the tendency of monomers to link via C−N=C groups, despite the induced stress. Finally, we comment on passing that since C‐NH‐C groups accommodate rotations better, and the XPS signal proves that these groups persist especially at lower temperatures, it is likely that these are related to the images where a curved configuration has been observed (e.g. Figure 3).

We have also investigated, from a theoretical perspective, the most viable coupling mechanism that could lead to the on‐surface formation of *m*‐PANI oligomers. We have employed the Climbing‐Image Nudged‐Elastic Band methodology to explore different scenarios for the dimerization of *p*‐AP on the Pt surface, including distinct initial molecular configurations and attacking sites.

**Figure 5:**
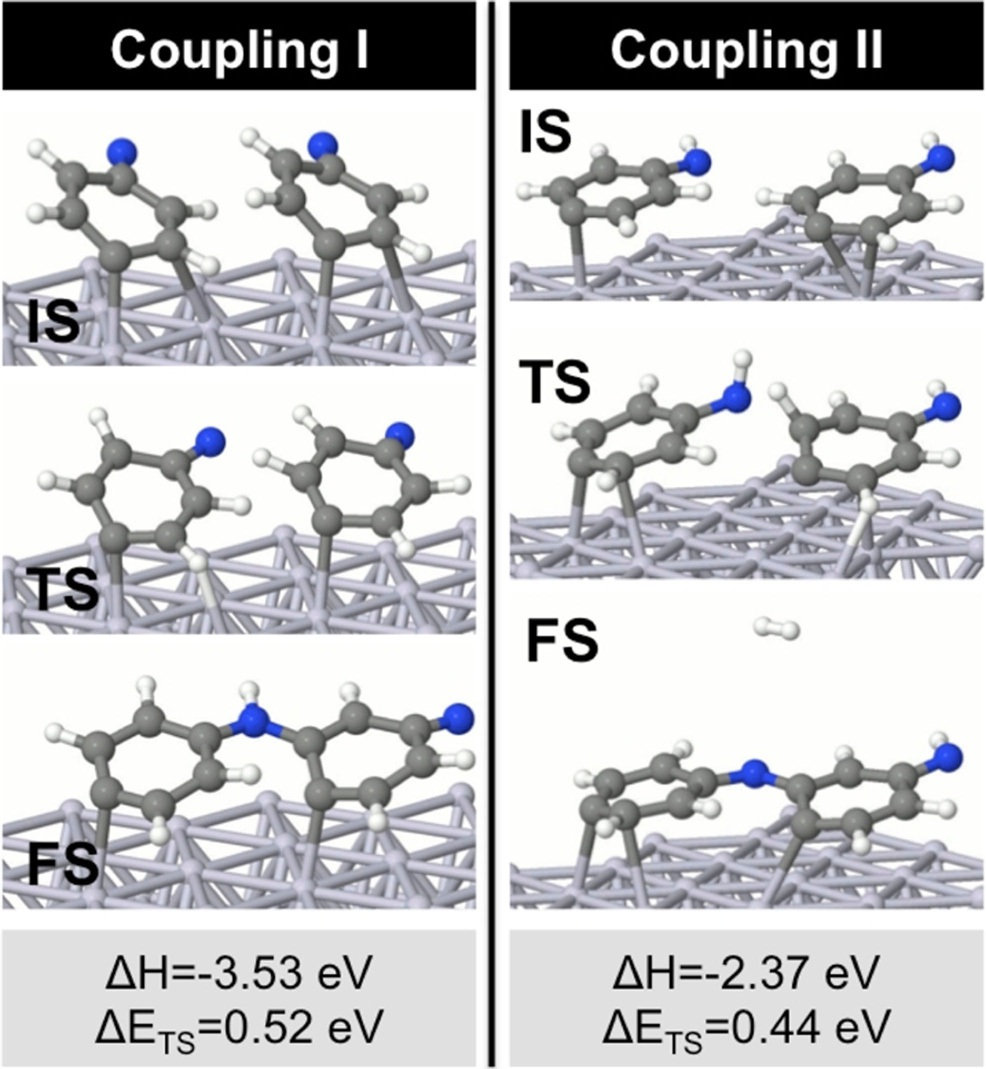
Pictorial view of the initial (IS), transition (TS), and final (FS) states for the two most favourable dimerization couplings of two different initial configurations of the precursors via the same attacking meta‐site. This situation corresponds to the first step towards the formation of *m*‐PANI. Variation of enthalpy for the adsorbed species (Δ*H*=H_FS_−H_IS_) between the final and initial states and energy barrier of the transition state are shown for each coupling.

Figure 5 presents a pictorial view of the main results obtained for the two most favourable dimerization couplings taking place at positions **1** and **4** (other less favourable coupling reactions are reported in the Supporting Information). It is worthy to note that, in both cases, the initial molecular composition employed was that corresponding to the results obtained from XPS at the temperature of interest, that is, the precursor molecules are dehydroxylated and the amino group is either partially or fully oxidized. Coupling I corresponds to the dimerization between two C_6_H_4_N precursors anchored to the surface through ring position **5**. In this pathway, the C−H bond (site **4**) reacts with the C−N bond of the adjacent molecule to form a C‐NH‐C linkage with an enthalpy gain of −3.53 eV and a barrier for the transition state of Δ*E*_TS_=0.52 eV. Coupling II shows the dimerization between two C_6_H_4_NH precursors. In this pathway, a C−H bond reacts with a C‐NH to form C‐N‐C bond and a gas‐phase H_2_ molecule, with an enthalpy gain and a barrier of −2.37 eV and 0.44 eV, respectively. Notice that these values correspond to species adsorbed on the surface and include the influence of the substrate on the polymerization energy. Thus, according to these results, the most energetically favourable dimer coupling would correspond to Coupling I. However, the very small difference in the energy barrier (Δ=+0.08 eV) implies that both coupling mechanisms could be kinetically viable on the surface; Coupling II leads to the growth of the proposed *m*‐PANI linear oligomers, whereas Coupling I could explain curved chains due to its enhanced flexibility towards small rotations.

As we have commented, even though the a priori natural linking positions between the *p*‐AP precursors would be the *para* positions (**1**–**5**, Scheme 1), this situation can be ruled out since the **1**–**5** coupling would result in a much larger repeat distance, above 5.2 Å. To grow on the Pt(111) surface, this large difference implies strong deformations in the oligomers that are not observed in the straight chains.

**Figure 6:**
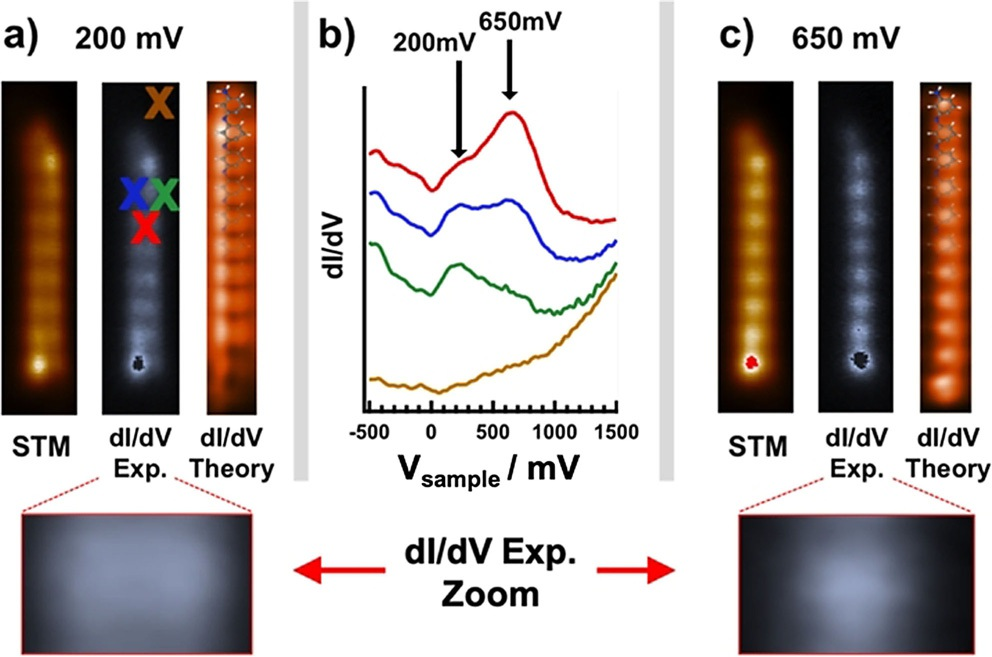
Electronic local density of states (LDOS) on a m‐PANI chain composed of 8 units, as extracted from STS measurements. a) from left to right: Tunneling current in constant height (regulation set point in the middle of the chain at 50 pA and −200 mV) recorded at 200 mV, Differential‐conductance (d*I*/d*V*) maps of unoccupied states (modulation 8.7 mV,) and theoretical d*I*/d*V* map on a 11 units oligomer. b) d*I*/d*V* spectra for the specific positions of the chain indicated as bold‐dots in Figure 6 a. The brown one corresponds to the typical LDOS of clean Pt(111). The green and the blue spectra are recorded at the edges of the chain and the red one represents the electronic states at the center of the chain. c) same as panel (a), but recorded at 650 mV. Bottom panel shows a detail of the d*I*/d*V* map to emphasize the double lobe structure for low voltages (left side) and the single lobe for higher values (right side).

Now that we have determined the nature of the so‐formed oligomers, we have turned our attention to their electronic local density of states (LDOS) as probed by d*I*/d*V* maps (Figure 6 a–c) and single‐point d*I*/d*V* spectra (Figure 6 b). Figure 6 b a presents a set of single‐point spectra recorded at different positions along the oligomer chains, together with the curve corresponding to the surface, which is used as reference. It can be clearly observed that two unoccupied states are present at around 200 and 650 mV. In order to determine the spatial distribution of these states, constant height topography and differential‐conductance map images have been acquired at the maximum energy position of each and are presented in Figures 6 a and c. Interestingly, there is an evident change in the appearance of the oligomers. While at 200 mV they can be visualized as a double maximum, at 650 mV each unit is made of a single rounded feature located at the centre of the two protrusions present at 200 mV, in marked contrast to the elliptical appearance of the constant current image of Figure 1 b. Even more interesting, the simulations indicate that these maxima correspond to the tilted phenyl rings and not to the linking N atoms, which are closer to the surface. Furthermore, the state at 200 mV is not symmetric, but one lobe is higher than the other (Figure 6 a, lower links of the chain: left lobe stronger than right lobe) and as we move along the chain the situation is inverted (Figure 6 a, upper links of the chain: right lobe stronger than left lobe).

For comparison, we have computed differential‐conductance STM images (constant‐current mode) at 0.1 nA and 200±25 and 700±25 mV on an 11‐link oligomer. Figure 6 a and c show the good agreement of both images with the experimental maps. Additionally, we have computed the Projected Density of States (PDOS) on a 5‐unit *m*‐PANI oligomer on Pt(111) (see details in supporting information). Albeit difficult because of the hybridization, the single‐lobe state at 650 mV seems to originate from a pronounced feature in the PDOS profile arising between 650 and 800 mV. Similarly, the two‐lobe state at 200 mV, which also has a reflection in the PDOS profile, can be associated with the LUMO of the oligomer.

Our theoretical calculations indicate that these oligomers, albeit lacking one hydrogen atom in the repeat units, present ferromagnetic properties.[^9^, ^10^] However, electronic hybridization with the substrate quenches the ferromagnetism when the oligomer is on the surface (see supplementary information).

The above‐described mechanism is based on a sequence of different dehydrogenation reactions in amine moieties and carbon rings, which have been previously described to take place stepwise at different temperatures on different surfaces.[^31^, ^48^, ^49^] Moreover, the proposed mechanism, confirmed by DFT calculations, explains why coupling occurs at a different site than the radical formation, as one could expect a priori. We show that although *para*‐precursors are used the bonding with the surface changes the coupling position resulting in a *meta*‐coupling of the chains.[^29^, ^32^, ^33^]

## Conclusion

We report an unprecedented on‐surface driven formal Michael‐coupling leading to the formation of *meta*‐polyaniline oligomers on Pt(111) surfaces directly from *p*‐aminophenol precursors, which we investigate using advanced scanning probe microscopies and electron spectroscopies, combined with theoretical methods. We show that the *para*‐aminophenol molecules adsorbed on Pt(111) at room temperature are reduced to *p*‐quinone imines and adopt an appropriate spatial position on the surface that promotes a temperature activated coupling leading to the formation of *m*‐PANI metal‐oligomers. The mechanism has been rationalized by first principles calculations that suggest only a small energy barrier for the process. The *m*‐PANI oligomers are incommensurate with respect to the Pt surface. As the molecule grows adding more repeat units, the accumulated mismatch leads to an important stress increase that limits the number of repeat units in the oligomer chains. The oligomers present well‐defined unoccupied electronic states at 200 and 650 mV that are spatially localized on the carbon rings. The reaction pathway proposed here is unviable in solution chemistry and it presents a high directionality that may be used for a rational synthesis of novel nanostructures of interest in the emerging fields of nanomagnetic materials and spintronics.

## Conflict of interest

The authors declare no conflict of interest.

## Supporting information

As a service to our authors and readers, this journal provides supporting information supplied by the authors. Such materials are peer reviewed and may be re‐organized for online delivery, but are not copy‐edited or typeset. Technical support issues arising from supporting information (other than missing files) should be addressed to the authors.

Supplementary
