# Supplementary material for: On‐Surface Driven Formal Michael Addition Produces m‐Polyaniline Oligomers on Pt(111)
Source: Angew Chem Int Ed Engl. 2020 Oct 12;59(51):23220–7. doi: 10.1002/anie.202009863 (PMC7116460; doi:10.1002/anie.202009863)
Supplement: Supplementary file 1 — Supplementary [file ANIE-59-23220-s001.pdf]

Angewandte Chemie
Eine Zeitschrift der Gesellschaft Deutscher Chemiker

Supporting Information

On-Surface Driven Formal Michael Addition Produces *m*-Polyaniline Oligomers on Pt(111)

Nerea Ruiz del Árbol, Carlos Sánchez-Sánchez, Gonzalo Otero-Irurueta, José I. Martínez, Pedro L. de Andrés, Ana C. Gómez-Herrero, Pablo Merino, Marten Piantek, David Serrate, Paolo Lacovig, Silvano Lizzit, José Alemán, Gary J. Ellis, María F. López, and José A. Martín-Gago**

anie_202009863_sm_miscellaneous_information.pdf

Table of Contents

1.- Experimental and theoretical procedures

2.- Alternative m-PANI models studied by DFT

3.- Nucleation mechanism towards the m-PANI chain polymerization

4.- Density of electronic states of the m-PANI/Pt(111) system

5.- Some theoretical insights into the magnetic properties

6.- nc-AFM calculations

7.- STM evolution with temperature

8.- XPS intensity analysis

9.- Supplementary references

1.- Experimental and theoretical Procedures

p-AP molecules (Sigma-Aldrich, 99% purity) were thoroughly outgassed and then spontaneously sublimated at room temperature from a crucible in the ultra-high vacuum (UHV) systems. The base pressure during evaporation was in the 10^{-9} mbar range and typical doses were in the order of 10 L. The Pt(111) surface was prepared by sputtering-annealing cycles. The first cycle was made under oxygen atmosphere (P_{O2} =1.10^{-6} mbar). Microscopy images were taken at 4.2 K and in a base pressure < 10^{-10} mbar. Scanning tunnelling microscopy and spectroscopy (STM and STS) measurements were performed in a SPECS Joule Thompson microscope with a sample bias convention and a modulation amplitude of 8.7 mV rms at 913 Hz. Non-contact atomic force microscopy (nc-AFM) experiments were performed in an Omicron LT microscope using a qPlus sensor in the frequency modulated mode with an oscillation amplitude of approximately 100 pm and a resonance frequency of 23.37 kHz

High-resolution fast x-ray photoelectron spectroscopy (XPS) measurements were performed *in situ* at the SuperESCA beamline of the Elettra synchrotron radiation facility in Trieste, Italy [S1]. The experimental chamber is equipped with a 150 mm Phoibos hemispherical electron energy analyser (SPECS GmbH), provided with a home-made delay line detector. The high-resolution core level spectra were measured in the normal emission configuration keeping the sample at 120K. C 1s and N 1s core levels were recorded at 480 eV of photon energy while O 1s was measured at 650 eV. The overall resolution was always better than 100 meV. For each spectrum, the binding energy scale was aligned to the Fermi level of the platinum substrate. Temperature programmed fast XPS measurements were performed during sample heating up to 1025 K at a programmed rate of 0.5 K /s; the average acquisition rate was about 12 seconds per spectrum.

Ab initio calculations for structural optimization, electronic structure properties and transition-state energy barriers, have been carried out by Density Functional Theory (DFT) as implemented in the plane-wave QUANTUM ESPRESSO simulation package [S2]. One-electron wave-functions are expanded in a plane-waves basis with energy cutoffs of 450 and 550 eV for the kinetic energy and the electronic density, respectively. Exchange and Correlation (XC) have been computed in the revised version of the generalized gradient corrected approximation (GGA) of Perdew–Burke–Ernzerhof (rPBE) [S3]. Rabe–Rappe–Kaxiras–Joannopoulos (RRKJ) ultrasoft pseudopotentials [S4, S5] have been adopted to model the ion–electron interaction. In all the calculations, the Brillouin zone (BZ) has have been sampled using Monkhorst–Pack grids [S6]. The van der Waals (vdW) interaction has been included via an empirical vdW R^{-6} correction (DFT+D3) [S7].

Atomic positions have been optimized using a conjugate gradient minimization scheme until the maximum force on any atom was lower than 0.02 eV/Å. The Fermi level was smeared out using the Methfessel–Paxton approach [S8] with a Gaussian width of 0.01 eV, and all energies were extrapolated to T=0 K. Self-consistency in the electron density to a precision in the total energy better than 10^{-6} eV was achieved.

Transition state geometries and energy barriers have been obtained with the Climbing-Image Nudged-Elastic Band method (CI-NEB) [S9], as implemented in QUANTUM ESPRESSO, using 12 intermediate images with a convergence threshold for the norm of the force orthogonal to the reaction path of 0.05 eV/Å.

In the next section, we argue that a comparison between experimental and theoretical distances is meaningful and allows us to discriminate different models. To support this hypothesis, we first benchmark our theoretical formalism by looking at distances and angles for two reference systems: (i) bulk Pt and (ii) the aniline molecule ($C_6H_5NH_2$) [S10]. Firstly, full optimization of the face-centred-cubic unit cell for Pt yields the correct symmetry group (correct angles) and, a lattice parameter of 3.93 Å, with residual stress better than 0.01 GPa, which compared with the experimental value of 3.912 Å yields an accuracy better than 0.5%. Secondly, we determine by full optimization of the aniline molecule <d(C-C)> = 1.397 Å (0.1%), <d(C-N)=1.394 Å (1%), and C-C-C angle at the N

position of 118.5º (0.4%). Therefore, the accuracy of this formalism determining distances can be assumed to be better than 1%, which allows us to discard theoretical models where lengths do not agree with experiments within that error bar.

Keldish-Green's functions formalism was used for simulation of the STM images [S10].

Results and Discussion

2.- Alternative *m*-PANI Models studied by DFT

Firstly, we have considered the formation of *p*-AP chains linked via *para* positions. None of the simulations produced a viable configuration. For all cases, monomer to monomer distances are above 5.2 Å for planar chains (the gas-phase *p*-polyaniline configuration), which is not compatible with the 4.6 Å distance experimentally observed unless the chain is deformed beyond that, impeded by steric requirements and that add a significant cost in energy.

To justify configuration I of Figure S1 as the most favourable model, DFT calculations were performed on geometries that would, in principle, also agree with the experimental evidence. In Figure S1, we report the structure and the most representative distances of the proposed model (Configuration I; top-left), and three alternatives for meta-PANI 4-monomer chains (configurations II-IV). The inter-monomer distance for the preferred configuration (Configuration I) adopts values between 4.62 and 4.68 Å, which agree well with the distance between monomers deduced from STM experiments, while the distance between Pt atoms on the surface along the direction of growth is 4.81 Å. Therefore, such a configuration does not commensurate with the substrate and it accumulates stress as it grows. In this configuration, distances between the bottommost C atoms on the chain and the first Pt atom of the surface are in the range of 2.0 to 2.1 Å. The adsorption energy per monomer is -1.36 eV and the polymerization energy (gas phase) is -5.32 eV.

The top-right model in Figure S1 (Configuration II) is a variant of Configuration I but with H atoms attached to the linking N atoms. In this case, the inter-monomer distance expands to 4.83 Å, according to the decreased bond order of the C—N—C moiety in the presence of H. Thus, this configuration commensurates better with the Pt-Pt distances on the surface. However, this model is not favoured because (i) the decrease in the energy of polymerization (Δ=5.3-3.9=+1.4 eV) is not sufficiently compensated by the increase in the interaction with the substrate (Δ=1.3-1.95=-0.65 eV) and, (ii) there is a noticeable discrepancy with the experimental distance between units (we have argued that such a comparison is possible due to the accuracy of our calculations, proved in relevant benchmark systems).

In the bottom-left model (Configuration III), we have attached H atoms to C atoms interacting closely with Pt atoms on the surface. This situation may arise by adsorption of residual H atoms from the surface. The inter-monomer distance is 4.68 Å, similar to that obtained in the first model but the average distance between the bottommost C atoms of the chain and the topmost Pt atom of the surface has increased up to values between 2.15 and 2.3 Å, evidencing a slight decoupling from the surface. Although presenting the same periodicity, this model will be less favourable than Configuration I as the energy of polymerization is lower (5.32-2.35=+2.97 eV) and the adsorption energy per monomer has increased to -1.28 eV. Additionally, this configuration will require an additional step consisting in the incorporation of H atoms to the structure after its formation, a step which presents a barrier of 0.87 eV (XPS data of Figure 2 indicate that C–O bonds are cleaved at oligomer formation temperature).

Finally, the bottom-right model (Configuration IV) includes H atoms attached to the C atoms linked to the surface and H atoms attached to the C atoms linking N atoms. The inter-monomer distance is 4.83 Å and the average distance between the bottommost C atoms of the chain and the topmost Pt atom of the surface varies between 2.15 and 2.3 Å. The energy of polymerization has significantly reduced with respect to configuration I (5.32-1.46=+3.86 eV) and the adsorption energy per monomer goes to -1.53 eV. As it happens in Configuration II, Configuration IV can be ruled out attending to periodicity and energetics arguments.

Therefore, we conclude from our DFT analysis that our first model best represents the experiments.

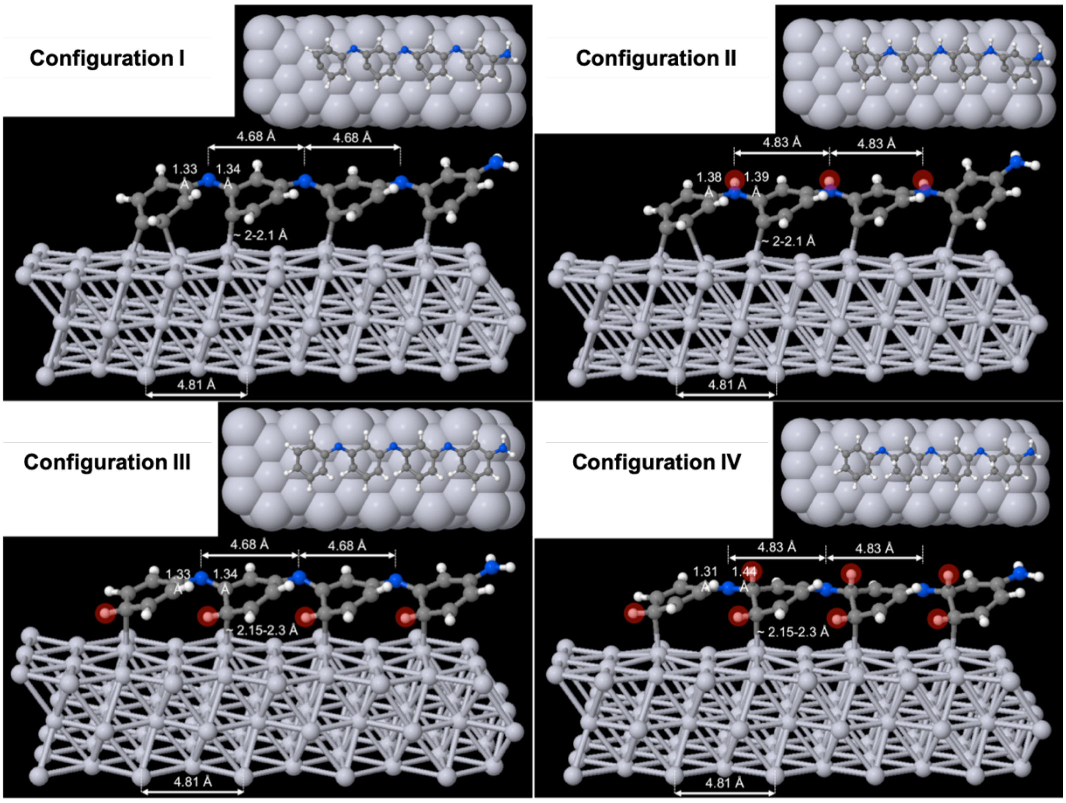

Figure S1. Structure and most representative distances computed by DFT of the proposed model and three alternative models of an m-PANI 4 units chain.

3.- Nucleation mechanism towards the m-PANI chain polymerization

Next, we turn our attention to the kinetics of the polymerization process. As commented above, we use the Climbing-image Nudged-elastic Band method (CI-NEB) [S11], as implemented in the QUANTUM ESPRESSO simulation package [S2]. A slab formed by three Pt(111) layers has been used to compute kinetic barriers. The two bottommost layers are fixed into ideal bulk positions and a 15 Å vacuum along the direction perpendicular to the surface is used to minimize the interaction between neighbouring unit cells. The minimum energy path so obtained yields the enthalpy variation between the initial and final state, the transition state structure, and the energy barrier at the transition state. This methodology has recently provided very successful results in the search of transition states and their associated energy barriers in similar related on-surface reactions [S12].

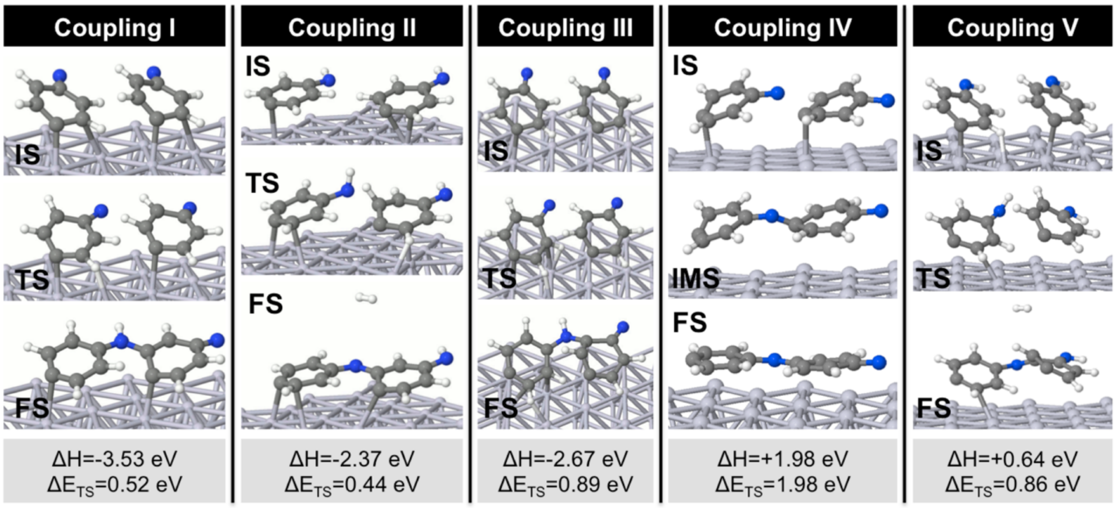

Figure S2. Pictorial view of the initial, transition and final state for five dimerization couplings of different precursors via different sites (Couplings I-V) towards the formation of dimers as origin of the nucleation in the polymerization of the one-dimensional chains. Variation of enthalpy $\Delta H=H_{FS}-H_{IS}$ between the final and initial states and energy barrier of the transition state are shown for each coupling.

In Figure S2, we report the main results for the coupling of different precursors via different sites (Couplings I-V) towards the formation of a dimer, as the first step to form a polymer. We show the initial (IS), final (FS) and transition states (TS) for all the coupling reactions, as well as the variation of enthalpy $\Delta H=H_{FS}-H_{IS}$ between the final and initial states and the kinetic barrier, ΔE_{TS}, except for cases IV and V, where the process is endothermic because the formation of the dimer is not enough to compensate the reduced interaction with the surface. Next, we describe in more detail each of the cases:

Coupling I. It shows the dimerization between two C_6H_4N precursors anchored on the surface and coupling via a *meta*- site, leaving a hydrogen atom attached to the N atom. In this reaction, a C—H bond breaks to form new C—N and N—H bonds, which yields a variation of enthalpy in favour of the product of -3.53 eV and a ΔE_{TS}=0.52 eV.

Coupling II. It shows the dimerization between two C_6H_4NH precursors again coupling via a *meta*- site and leading to the formation of a H_2 molecule. In this reaction, a C—H and a N—H bond break to form a new C—N bond and a gas-phase H_2 molecule, which yields a variation of enthalpy in favour of the product of -2.37 eV and a lower ΔE_{TS}=0.44 eV.

Coupling III. It shows the dimerization between two C_6H_4N precursors (similar to coupling I), but in this case the coupling takes place via an *ortho*- site, leaving again a hydrogen atom attached to the N atom. In this coupling reaction, a C—H bond breaks to form new C—N and N—H bonds. Nevertheless, this reaction induces a slight distortion of one of the rings in the final state, which yields a variation of enthalpy in favour of the product lower than in the coupling I case (-2.67 eV) and with a significantly higher barrier of ΔE_{TS}=0.89 eV.

Coupling IV. It shows the dimerization between two C_6H_4N precursors anchored on the surface (similar to coupling I and III), but in this case the coupling takes place via a *para*- site. In this coupling reaction, two C—Pt bonds break to achieve the final physisorbed state on the surface, forming a new C—N bond. This reaction flattens the final state and decouples it from the surface,

which yields a variation of enthalpy in favour of the on-surface reactants of +1.98 eV, which indicates that this reaction is clearly endothermic and unviable energetically.

Coupling V. It shows the dimerization between two C_6H_4NH precursors coupling via an *ortho*- site, leading to the formation of a H_2 molecule (similarly to coupling II). In this reaction, a C—H, a N—H, and two C—Pt bonds break forming a new C—N bond and a gas-phase H_2 molecule. Nonetheless, the strong distortion induced in the final state and its partial on-surface decoupling yields a variation of enthalpy once again in favour of the on-surface reactants of +0.64 eV, which indicates that this reaction is also clearly endothermic and unviable energetically.

According to these results, we conclude that Coupling II is the kinetically favoured mechanism to form the dimer, with a variation of enthalpy of -2.37 eV and the lowest energy barrier at the transition state of 0.44 eV, due partly to the formation of a H_2 molecule.

4.- Density of electronic states and magnetization of the *m*-PANI/Pt(111) system

We have computed the Density of States (DOS) for the optimized model of a 5-unit *m*-PANI chain on Pt(111). Figure S3 shows the contributions to the Projected Density of Electronic States (PDOS) of the different parts of a 5-unit *m*-PANI chain as a function of the energy (in eV), referred to the Fermi level onto the whole 5-unit fragment (blue line). For unoccupied states, it is possible to observe two peaks at around 0.2 eV and 0.7 eV, which can be paired with the two peaks observed in the STS spectrum at very similar energies, and for which STM images have been experimentally acquired and simulated.

Given the broad PDOS profiles obtained, it is not simple to identify the origin of these peaks. For this reason, we have computed the PDOS profiles projected into (i) all the C atoms in the chain (black line), (ii) the five bottommost C atoms binding to the Pt surface (red line) and, (iii) the N atoms (green line). From these calculations, we conclude that the two peaks are visible on all these projections, which indicate that these states are (i) located in the *m*-PANI fragment and, (ii) they have a delocalized character, exhibiting an electronic imprint in all the atoms involved in the fragment chain.

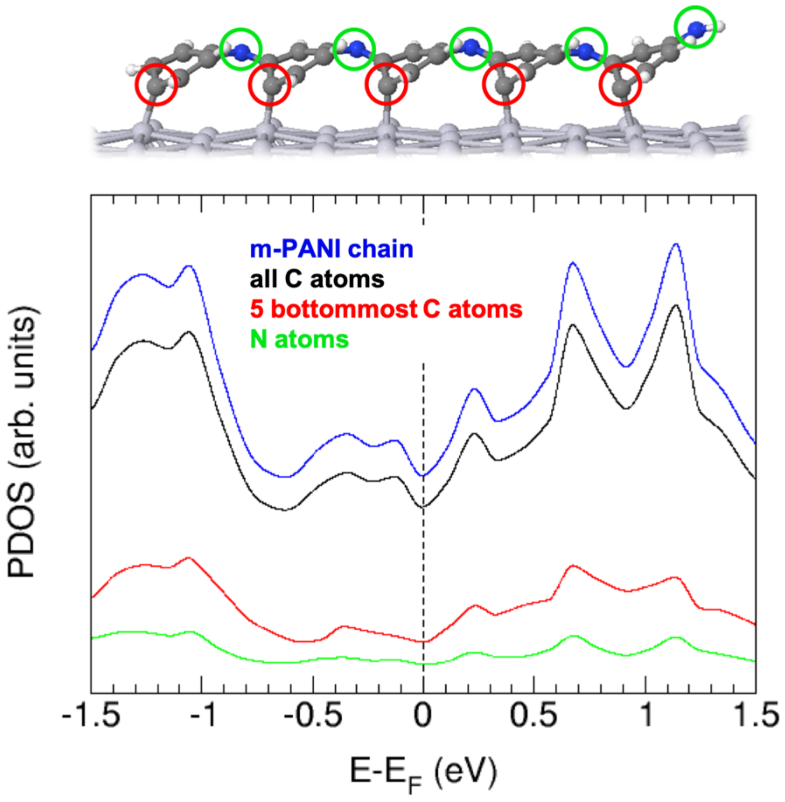

Figure S3. DFT-computed projected density of electronic states (PDOS) profiles, as a function of the energy (in eV) referred to the Fermi level, onto: the whole 5-units fragment (blue line), all the C atoms in the chain (black line), the 5 bottommost C atoms binding the Pt surface (red line), and the N atoms (green line). Pictorial side view of the optimized structure is also shown.

5.- Some theoretical insights into the magnetic properties

We have explored, from a theoretical perspective, the magnetic properties of *m*-PANI fragments with even and odd number of monomers; in particular the 4- and 5-unit oligomers in both their free and on-surface forms. Our spin-polarized DFT results reveal that free oligomers show a tendency to form an antiferromagnetic state, as can be seen by comparing the total integrated magnetization and the integral corresponding to its absolute value (Figures S4a and S4b). The 4-unit oligomer (Figure S4a) shows a total magnetization of M_{total} = 0.9 μ_B (being μ_B the Bohr's magneton), while the absolute value gets to $|M_{abs}|$ = 4.9 μ_B. A comparison with a 5-unit oligomer (Figure S4b) shows that the effect persists with M_{total} = 0.4 μ_B and $|M_{abs}|$ = 4.9 μ_B. Figure S4 also shows the atomic magnetic moment in each atom (in μ_B units) for each oligomer, their total density of states, and the spin-magnetization, resolved in energy (as the difference between the DOS spin-up and spin-down channels). The Bader analysis of the spin distribution over the atoms shows a complex pattern where the chain running along the N atoms tends to form a ferromagnetic alignment with low and quite constant values of the atomic magnetic moments, while spins along the lower chain tends to show a polarization (with an opposite alignment in both free oligomers, which may be explained in terms of an odd-even effect) with higher values of the atomic magnetic moments.

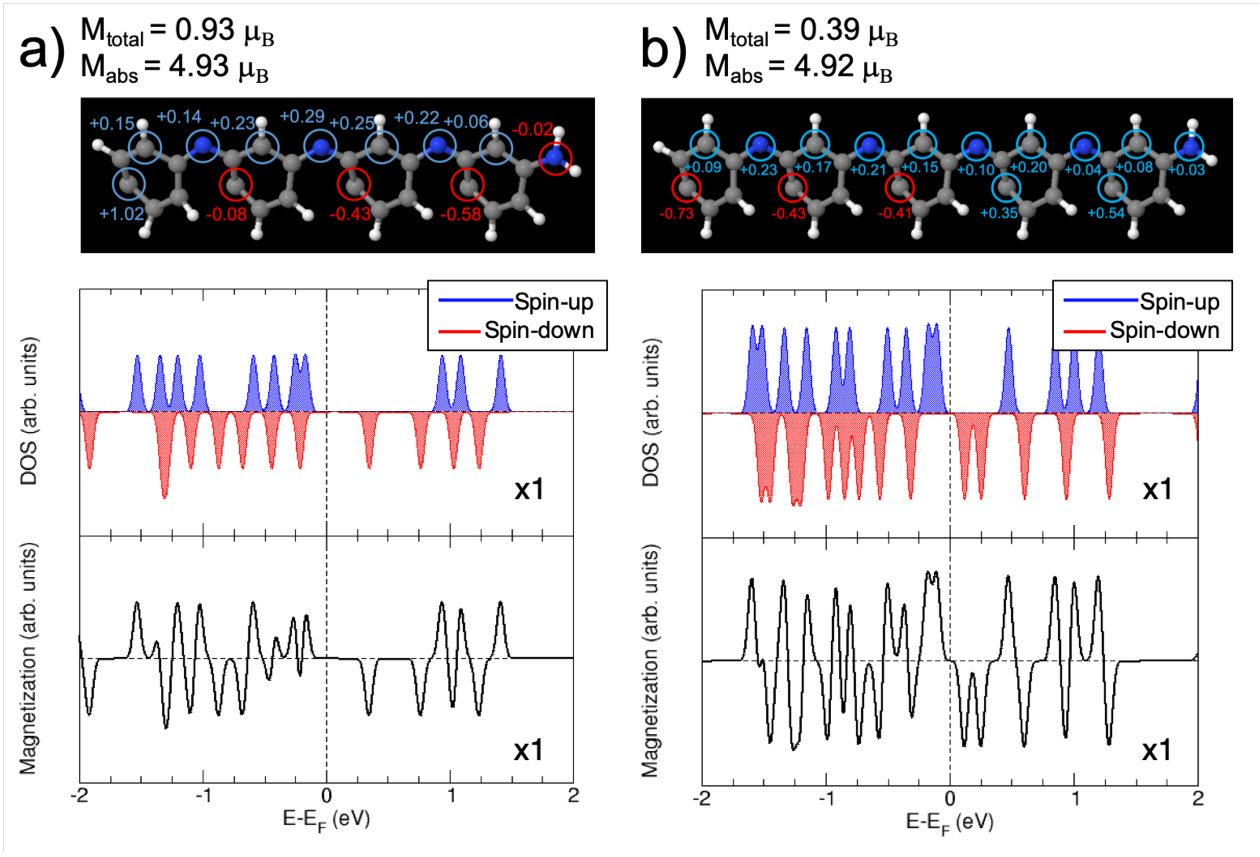

Figure S4. Total integrated (Mtotal) and absolute (Mabs) magnetizations (in μ_B units), Bader atomic moments (in μ_B units), and spin-resolved density of states and spin-magnetization (in arb. units) as a function of the energy (in eV) referred to the Fermi energy for the free: a) 4-unit oligomer, and b) 5-unit oligomer. Y-scale in DOS and Magnetization graphs is the same.

However, any trace of magnetism is quenched for the oligomers adsorbed on the Pt(111) surface. The 5-unit oligomer always converged to a non-magnetic state, irrespective of the initial spin state considered. The 4-unit oligomer also converged to a non-magnetic state for its global equilibrium minimum state. Nonetheless, two metastable configurations, with energies +0.02 and +0.06 eV higher with respect to the equilibrium configuration, have been found. These metastable configurations exhibit a small remnant magnetic moment and their total energies indicate that they should be accessible by thermal fluctuations. Interestingly, we notice that the small remaining spin distribution is located on the Pt atoms and not on the polyaniline-like chain. Figure S5 shows a summary of the results for these metastable configurations for the on-surface 4-unit oligomer.

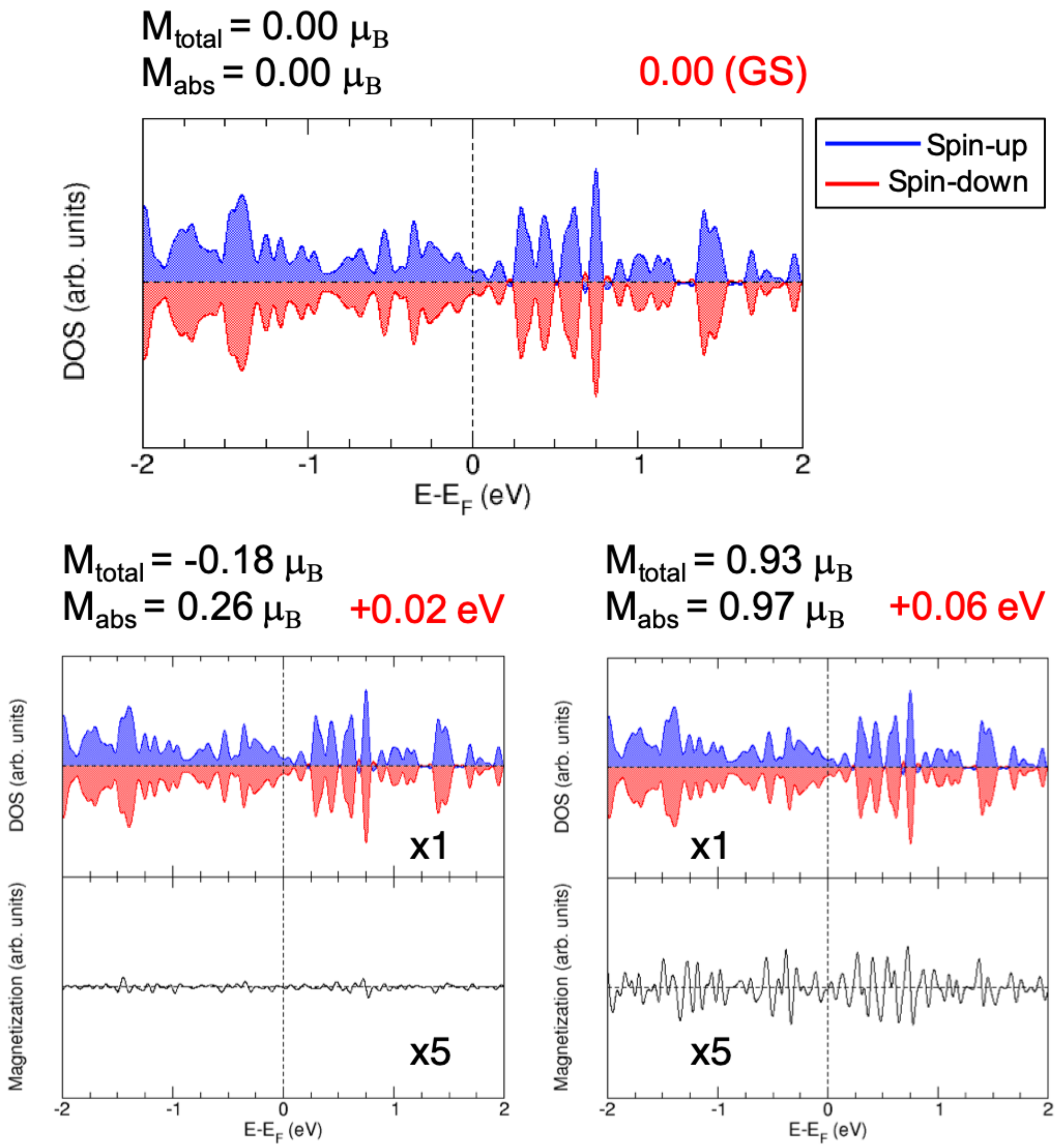

Figure S5. Total integrated (Mtotal) and absolute (Mabs) magnetizations (in μ_B units), and spin-resolved density of states and spin-magnetization (in arb. units) as a function of the energy (in eV) referred to the Fermi energy for the three different spin configurations found for the on-surface 4-unit oligomer, also indicating the difference in total energy w.r.t. the ground-state (GS). For a better visualization, Y-scale in the Magnetization graphs has been multiplied by a factor x5 w.r.t. the corresponding DOS ones.

A word of caution is in order about these findings: akin to our experimental limitations, a good characterization of the magnetic state would require tools beyond our current use of DFT, which would not be feasible because of the large number of atoms involved. In particular, a better description of e-—e- (correlation) and e-—ph interactions. On the other hand, it seems clear that the oligomer adsorbed on the surface shows a weaker magnetic state, which requires even finer tools to obtain a reliable characterization of the magnetic properties.

6.- Nc-AFM calculations

Figure S6 shows the experimental and simulated nc-AFM images [S13, S14] of an 11 unit PANI oligomer, with the DFT model superimposed on the image. The similarity of both images suggests that the structural model presented in the study is correct.

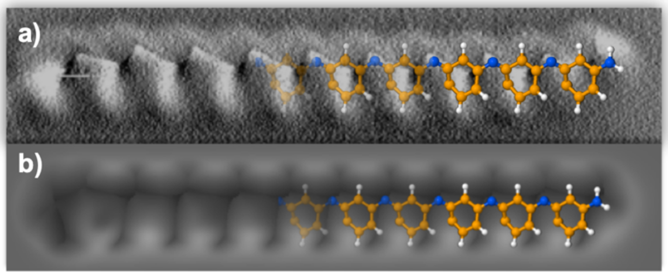

Figure S6. a) Experimental Nc-AFM image of figures 1d and 4b (10-unit m-PANI chain on Pt(111)) with the DFT optimized model superimposed on the image. b) Simulated high-resolution AFM (Δf) image of the 11-unit m-PANI chain on Pt(111) of Fig. 4a at a tip-sample distance of 3 Å by using an effective stiffness of k=0.25 Nm-1 and a s-like monopole charge typical CO tip model negatively charged (Qs=-0.1 e-A2). Simulations of the high-resolution AFM images have been undertaken by using the ProbeParticleModel simulation package developed by P. Jelínek and coworkers [S13, S14].

7.-Evolution of the molecular structures with temperature

Figure S7 shows the evolution of the molecular structures upon increasing temperature, as observed by STM. The first STM image was recorded at room temperature and individual molecules aligned along the main symmetry direction of the surface can be distinguished. This image corresponds to range B of Figure 2 and the molecules form a self-assembled layer of chemically modified *p*-Ap. XPS core level analysis indicates that the alcohol has dehydrogenated as well as the primary amine groups, which have transformed into secondary amines. As the temperature is increased, in range C, (panels b and c), the relative content of straight oligomers increases with respect to the short ones, in good agreement with the transformation from secondary to tertiary amines depicted by the XPS data of Figure 3, although both coexist on the surface. This ratio increases very slowly above 600 K. Finally, as the temperature reaches about 800 K, graphitization takes place and small, brighter patches appear distributed on the surface (marked with an arrow in Figure S7). At this temperature, some nitrogen atoms start to be removed (see table S1 and fast XPS of Figure2) while others combine with remaining C atoms from the rings and fuse together to form N-doped graphene, as evidenced by XPS and reported for other molecules on Platinum after high temperature annealing [S15].

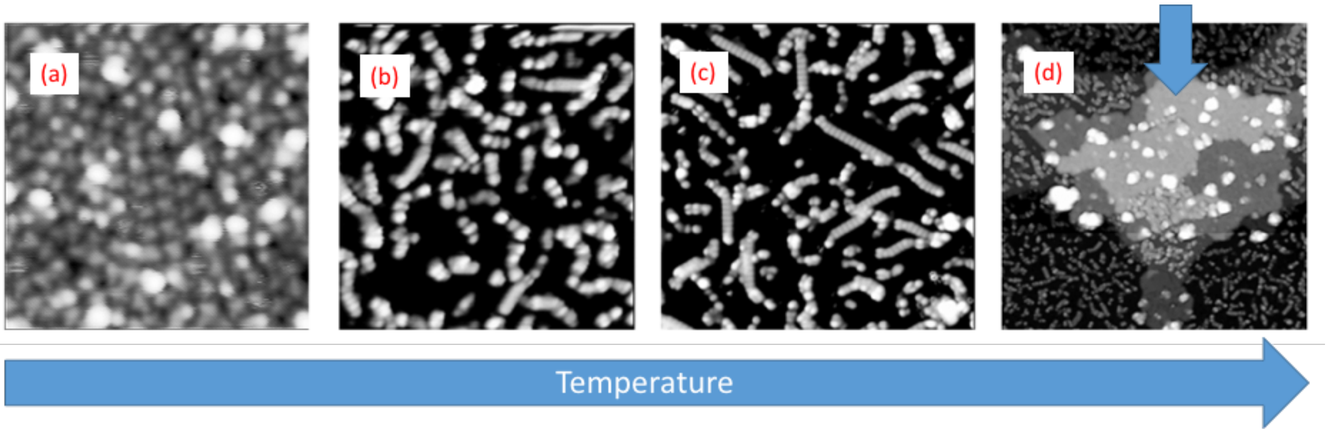

Figure S7. Serie of STM images with increasing temperature: a) 7 ×7) nm^2; It = 0.04 nA; Vb = 1.2 V. b) (25 × 25) nm^2; It = 0.05 nA; Vb = 1 V. c). (25 × 25) nm^2; It = 20 pA; Vb = 1.2 V d) (75 × 75) nm^2; It = 50 pA; Vb = -200 mV. A graphene island, indicated with an arrow, is seen in the image.

To better show the gradual conversion of C-NH-C to C-N=C bonds, we have carried out a STM statistical analysis of the evolution of curved *vs* straight chains as a function of sample temperature. Figure S8 shows the corresponding table including the percentage of C-NH-C and C-N=C bonds at each temperature, together with the total number of events considered for each temperature. As it can be observed, although the absolute values are not identical, above all at lower temperatures, the trend is clear and in agreement with the complementary XPS results presented in Figure 3. A cross-over of both components of about 500K is evident from this comparison. It should be noted that the errors in the STM analysis are larger than in XPS due to the presence of undefined structures that were not assigned to any of the cases.

Table S1. Statistical analysis of the thermal evolution of C-NH-C and C-N=C bonds as obtained from STM. A total number of 563 events from 12 STM images have been considered in this analysis.

Temperature [K]	C-NH-C [%]-Curved chains	C-N=C [%]-Linear chains	# Events
425	58	42	156
525	46	54	170
625	35	65	237

8.- XPS intensity analysis

Figure S9 shows the evolution of the XPS core level line-shape and intensity of the O 1s, N 1s, and C 1s peaks upon increasing temperature. By integrating these peaks we have obtained the data shown in table S1. These data clearly show that the *p*-AP molecules arrive at the surface with the correct C:N:O stoichiometry, which is maintained up to 525K. In the temperature region where chains are formed (region C in Figure 2), the C–O bond is cleaved and oxygen disappears from the molecular structure, whereas the concentration of N atoms is maintained in the oligomer.

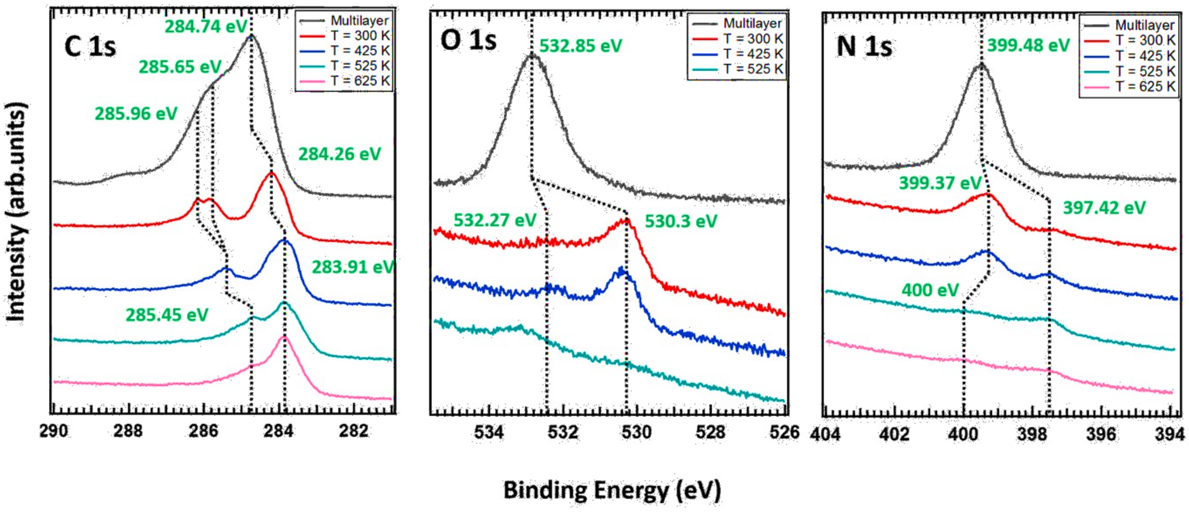

***Figure S9**: Serie of high resolution XPS spectra recorded at different temperatures. Photon energies: O1s recorded at 620 eV, N1s and C1s at 480 eV.*

Table S2: Normalized intensity ratio for the total area of the elements in previous figure for the most important temperatures. The last row shows the stoichiometry derived from these values. Peaks were normalized to the flux and cross section at their respective photon energies.

	Multilayer	*300 K*	*425 K*	*525 K*
N/C	0.20	0.16	0.16	0.15
O/C	0.16	0.17	0.20	0
N/C/O	1.2 / 6 /1.0	1.0 / 6 /1.0	1.0 / 6 /1.2	0.9 / 6/ 0

9.- Supplementary references

[S1] A. Baraldi, G. Comelli, S. Lizzit, D. Cocco, G. Paolucci, R. Rosei, Surf. Sci. **1996**, *367*, L67–L72.
[S2] P. Giannozzi, et al., J. Phys.: Condens. Matter. **2009**, *21*, 395502.
[S3] J. P. Perdew, K. Burke, M. Ernzerhof, Phys. Rev. Lett. **1996**, *77*, 3865–3868.
[S4] A. M. Rappe, K. M. Rabe, E. Kaxiras, J. D. Joannopoulos, Phys. Rev. B **1990**, *41*, 1227–1230.
[S5] N. Mounet, N. Marzari, Phys. Rev. B - Condens. Matter Mater. Phys. **2005**, *71*, 1–14.
[S6] J. D. Pack, H. J. Monkhorst, Phys. Rev. B **1977**, *16*, 1748–1749.
[S7] S. Grimme, J. Antony, S. Ehrlich, and H. Krieg, *J. Chem. Phys.* **2010**, ***132***, 154104.
[S8] M. Methfessel, A. T. Paxton, Phys. Rev. B **1989**, *40*, 3616–3621.
[S9] G. Henkelman and H. Jónsson, J. Chem. Phys. **2000**, *113*, 9901.
[S10] J. M. Blanco, F. Flores, R. Pérez, *Progress in Surface Science* **2006**, *81*, 403–443.
[S11] G. Schultz, G. Portalone, F. Ramondo, et al., Struct. Chem. **1996**, *7*, 59–71.
[S12] C. Sánchez-Sánchez, et al., J. Amer. Chem. Soc. **2019**, *141*, 3550.
[S13] P. Hapala, G. Kichin, C. Wagner, F. Stefan Tautz, R. Temirov, and P. Jelínek, Mechanism of high-resolution STM/AFM imaging with functionalized tips, Phys. Rev. B **2014**, *90*, 085421.
[S14] P. Hapala, R. Temirov, F. Stefan Tautz, and P. Jelínek, Origin of High-Resolution IETS-STM Images of Organic Molecules with Functionalized Tips, Phys. Rev. Lett. **2014**, *113*, 226101.
[S15]. A. L. Pinardi, G. Biddau, K. van D. Ruit, G. Otero-Irurueta, S. Gardonio, S. Lizzit, R. Schennach, C. F. J. Flipse, M. F. López, J. Méndez, R. Pérez, J. A. Martín-Gago, *Nanotechnology* **2014**, *25*, 385602.

Author Contributions

N.R.dA. and C.S-S were involved in all experiments and main data analysis. J.I.M. and P.L.dA. performed the DFT calculations. G.O-I, A.G.-H. performed first STM experiments. P.M., M.P. and D.S. collaborated in the STM and nc-AFM experiments. P.L., S.L. and G.O-I contributed in the XPS measurements. J.A., G.J.E, C.S-S, M.F.L. and J.A.M-G developed the chemical interpretation of the work. All authors contributed to the writing of the manuscript. J.A.M-G. coordinated all the work.
